# Mathematical modelling of *Echinococcus multilocularis* abundance in foxes in Zurich, Switzerland

**DOI:** 10.1186/s13071-016-1951-1

**Published:** 2017-01-11

**Authors:** Belen Otero-Abad, Simon R. Rüegg, Daniel Hegglin, Peter Deplazes, Paul R. Torgerson

**Affiliations:** 1 Section for Veterinary Epidemiology, Vetsuisse Faculty, University of Zurich, Zurich, Switzerland; 2 Institute of Parasitology, Vetsuisse Faculty, University of Zurich, Zurich, Switzerland

**Keywords:** *Echinococcus multilocularis*, Alveolar echinococcosis, Epidemiology, Transmission, Mathematical modelling

## Abstract

**Background:**

In Europe, the red fox (*Vulpes vulpes*) is the main definitive host of *Echinococcus multilocularis*, the aetiological agent of a severe disease in humans called alveolar echinococcosis. The distribution of this zoonotic parasite among the fox population is remarkably aggregated with few heavily infected animals harbouring much of the parasite burdens and being responsible for most of the environmental parasitic egg contamination. Important research questions explored were: (i) spatial differences in parasite infection pressure related to the level of urbanization; (ii) temporal differences in parasite infection pressure in relation to time of the year; (iii) is herd immunity or an age-dependent infection pressure responsible for the observed parasite abundance; (iv) assuming *E. multilocularis* infection is a clumped process, how many parasites results from a regular infection insult.

**Methods:**

By developing and comparing different transmission models we characterised the spatio-temporal variation of the infection pressure, in terms of numbers of parasites that foxes acquired after exposure per unit time, in foxes in Zurich (Switzerland). These included the variations in infection pressure with age of fox and season and the possible regulating effect of herd immunity on parasite abundance.

**Results:**

The model fitting best to the observed data supported the existence of spatial and seasonal differences in infection pressure and the absence of parasite-induced host immunity. The periodic infection pressure had different amplitudes across urbanization zones with higher peaks during autumn and winter. In addition, the model indicated the existence of variations in infection pressure among age groups in foxes from the periurban zone.

**Conclusions:**

These heterogeneities in infection exposure have strong implications for the implementation of targeted control interventions to lower the intensity of environmental contamination with parasite eggs and, ultimately, the infection risk to humans.

**Electronic supplementary material:**

The online version of this article (doi:10.1186/s13071-016-1951-1) contains supplementary material, which is available to authorized users.

## Background

*Echinococcus multilocularis* is a zoonotic cestode present in large parts of the northern hemisphere. The parasite is sustained by a wildlife cycle with carnivores (mainly foxes) as definitive hosts and small mammals (mainly rodents) as intermediate hosts [1]. However, domestic dogs are believed to be an infection source for humans in Asia [2, 3]. Humans are accidental hosts that become infected through the ingestion of parasitic eggs excreted through the faeces of the infected canids [1]. The metacestode stage of this tapeworm causes chronic life-threatening alveolar echinococcosis (AE), which can have a high economic impact in highly endemic resource-poor settings [4]. The geographic distribution of *E. multilocularis* seems to be expanding and it is considered an emerging/re-emerging pathogen in many countries [5–8]. In Europe, high prevalences (23.9–57.3%) of *E. multilocularis* have been frequently reported in the red fox population (*Vulpes vulpes*) [9–11], which is increasingly colonising urban areas [12, 13]. In Zurich (Switzerland), the abundant availability of anthropogenic food seems to have contributed to the gradual increase of the urban fox population [14]. Moreover, the establishment of an *E. multilocularis* transmission cycle in the urban and periphery of Zurich is well documented [11, 15–17] as conditions appear to sustain high densities of foxes that support an active parasite life-cycle. These findings, along with the increasing incidence of human AE [18] have raised public health concerns and the demand to implement disease control strategies [15, 19].

Variations between individuals in their exposure and susceptibility to parasite infective stages result in the aggregated distribution of parasites within their hosts [20]. The distribution of *E. multilocularis* in foxes is also characteristically aggregated with most animals carrying low numbers of parasites whereas a few harbour thousands of them. The risk of developing human AE depends, among other factors, on the amount of infective eggs found in the environment and their accessibility to humans [21]. Due to the parasite aggregation, the degree of egg contamination in the environment depends greatly on a few highly infected animals [11, 15, 22]. However, as eggs can survive in the environment for some time, there may also be some contribution from less heavily infected foxes. Information on prevalence in foxes has been often used to characterize the infection risk for AE as its estimation is more reliable and straightforward than other epidemiological parameters [23]. However, there is not a clear correspondence between prevalence rates and parasite abundance in the fox population [24]. Hence, there are major limitations when using prevalence in foxes to describe the epidemiology of *E. multilocularis* infection [11]. The determination of parasite abundance in animal hosts can provide valuable information to optimize parasite control strategies. For instance, if there is evidence of spatial heterogeneities in *E. multilocularis* infection pressure, anthelmintic baits can be distributed in areas where superinfected animals are predicted to be in order to reduce more efficiently the environmental contamination of eggs and ultimately, human infection. A key epidemiological parameter to predict parasite abundance in the animal host is the infection pressure. The parasite burden in the definitive host depends on the number of infectious stages encountered per infection insult, meaning the number of viable protoscoleces contained in the hydatid cysts that the intermediate host carries. This study complements the results reported on the force of infection by a study on the mathematical modelling of *E. multilocularis* infection in foxes in Zurich [25]. There, the force of infection is defined as the number of exposures per unit time regardless of the quantity of parasites to which a fox is exposed [25].

The infection pressure cannot be estimated through direct observation; hence, we use mathematical models that allow inference on processes relevant to transmission as well as their quantification, in conjunction with field data. Besides the specific research question we want to address and the identification and incorporation of the epidemiological knowledge available, the selection of an appropriate model will depend on its ability to represent the available field data. The data for the present study consisted of parasite counts found in necropsied foxes collected in three different spatial zones within the municipality of Zurich [17]. Several studies have been carried out on *E. multilocularis* transmission in foxes in Switzerland providing an extensive prior knowledge for model construction and hypothesis formulation. Previous studies of *E. multilocularis* in Switzerland have shown that transmission dynamics in animal hosts are influenced by multiple interrelated factors that contribute to its spread [11, 17, 26–28]. Decreasing parasite prevalences along with the increasing level of urbanization have been reported in foxes in the two largest cities of Switzerland [11, 27, 28]. Special attention was brought to the intermediate areas between the rural and urban habitats where the proportion of *E. multilocularis* coproantigen-positive fox faeces was higher compared to the urban area [16]. These areas are believed to be heavily contaminated by infective eggs, and thus may represent *hot-spots* for human infection [15]. In addition, there is evidence of seasonal variation in parasite abundance in Swiss foxes, which has been found to be related with the age of the host [11, 17, 26]. In addition, juvenile foxes of less than one-year-old have frequently been reported bearing higher infection rates and parasite burdens [11, 17, 26, 27, 29]. The study quantifying the force of infection in *E. multilocularis* in foxes in Zurich, defined as the number of fox exposures to parasite infection (insults) per unit time, reported spatial and seasonal variations in incidence of exposure [25]. However, it did not address parasite abundance, which is important for the transmission dynamics. Here, we adapted existing transmission models describing the number of parasites depending on host age [30, 31] to estimate the spatio-temporal variation of the infection pressure. We aim to address further specific research questions: (i) are there spatial differences in parasite infection pressure related to the level of urbanization; (ii) are there temporal differences in parasite infection pressure in relation to time of the year; (iii) is herd immunity or an age-dependent infection pressure responsible for the observed parasite abundance; (iv) assuming *E. multilocularis* infection is a clumped process, how many parasites results from a regular infection insult.

## Methods

### Study data

The data used for this study was sourced from the necropsies of red foxes collected from January 1996 to April 2000 within the political community of Zurich as part of the Integrated Fox Project and before the implementation of an anthelmintic baiting study [11, 17]. The age of each fox was determined in years through dental examination [32] assuming all cubs were born on the first of April, as described previously [33]. In this study, we used the dates when the foxes were collected to estimate the approximate age in days of each fox. Foxes less than 1-year-old were classified as juveniles [34]. Each animal was further classified as coming from the periurban, border or urban zone, depending on where it was collected. The characteristics of each spatial zone have already been described in detail by Hegglin et al. [17]. The periurban zone refers to the external ring delimiting the city of Zurich and which mainly comprises a green belt of forests, fields, pastures, and meadows. The border area refers to the intermediate ring that contains residential areas, allotment gardens, cemeteries, sports fields, and public parks. The urban area refers to the center of the city encompassing much of the built-up zone.

For the analysis, we used *E. multilocularis* intestinal counts from 531 foxes aged up to 9 years old. Thus, the parasite biomass is the total number of parasites recovered from the foxes. The median age was less than 1-year-old in all zones. The group had an overall prevalence of 41.4% and a median abundance of 0 parasites (95% central range 0–10,488 parasites). All the data is provided in Additional file 1.

### Age-based abundance model

**Fig. 1:**
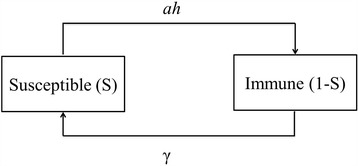
Graphical representation of the transmission model for *E. multilocularis* in animal hosts. The model represents the proportion of animals that develop immunity upon exposure to the infectious parasite stages at rate *ah* and the proportion that return to susceptibility at rate γ. Where *a* is the rate of acquisition of immunity, *h* is the infection pressure in parasites per year and γ is the rate of loss of parasite immunity

The association between parasite burden and age in foxes [11, 26, 27] suggested the use of an age-stratified SIR model originally developed by Roberts et al. [30]. It stratifies the host population into compartments that represent their infection and immune status at a particular age and the transition between states can be described by a set of ordinary differential equations. A schematic representation of the model is given in Fig. 1.

The model describes the variation in the proportion of animals susceptible to infection (equation 1) and the change of parasite abundance with respect to the host’s age *t* (equation 2).

1 $$ \frac{dS}{dt}\kern0.5em =\kern0.5em \gamma \kern0.5em -\kern0.5em \left(\gamma \kern0.5em +\kern0.5em ah\right)S $$

2 $$ \frac{dM}{dt}\kern0.5em =\kern0.5em hS\kern0.5em -\kern0.5em \mu M $$

where *S* is the proportion of susceptibles, *t* is the age of the host, *γ* is the rate of loss of immunity to parasites by foxes, *a* is the rate of acquisition of immunity, *h* is the infection pressure in number of parasites per year, *M* is the parasite abundance and *μ* is the parasite death rate (1/*μ* is the parasite life expectancy). The infection pressure, in the present report, is defined as the number of adult worms that would develop in the definitive host after parasite exposure in the absence of density-dependent constraints.

By adapting these equations, we attempt to answer our questions on *E. multilocularis* infection pressure and build models describing different plausible scenarios that might explain the parasite abundance observed in the foxes. As a result, a series of models assessing the existence of spatio-temporal and age-dependent variations in infection pressure were developed. The model parameters and descriptions are summarized in Tables 1 and 3, respectively.

**Table 1:** Description of the abundance model parameters for *E. multilocularis* in foxes in Zurich

Parameter	Description
*β*_*0*_	Baseline number of parasites of the infection pressure
*β*_*p*_	Amplitude of the infection pressure in the periurban zone
*β*_*b*_	Amplitude of the infection pressure in the border zone
*β*_*u*_	Amplitude of the infection pressure in the urban zone
*ϕ*_*p*_	Decrease parasite rate in the periurban zone
*a*	Rate of acquisition of immunity on exposure
*γ*	Rate of loss of immunity
*μ*	Parasite death rate

### Models assessing spatial differences in the infection pressure

The study area was divided into three spatial zones, periurban, border, and central urban covering 20%, 41%, and 39% of the study area respectively. Three different scenarios were considered: (i) the study area comprised just one spatial zone; (ii) the study area comprised two different spatial zones, the periurban and the suburban which includes the border and urban zones and; (iii) the study area comprised three different spatial zones, periurban, border and urban. The border and urban area were merged into one zone in the second scenario to consider the possibility of no spatial differences in infection pressure between both areas, as they are quite alike. These scenarios for different spatial zones were analyzed by using either 1 model with a single value for the infection pressure *h*, 2 different values for *h* for 2 zones and 3 values of *h* for 3 zones (in equations 1–4). Likewise, it was analyzed if there were potentially 1, 2, or 3 baseline infection pressure *β*_*0*_ between the zones (see equations 3 and 4)

### Models assessing time-dependent infection pressure

The evaluation of the time dependence of the infection pressure using host age as proxy for time resulted in three different functions (equation 3), where *β*_*0*_ represented the baseline number of parasites in a year and *β* the amplitude by which this baseline could vary according to a linear or periodic relationship. The models accounted for different baseline infection pressures and amplitudes for each of the three urbanization zones: periurban (*β*_*p*_), border (*β*_*b*_) and urban (*β*_*u*_). A log link function was implemented to ensure positive estimates of *β*_*0*_ and *β*, as previously described [25].

3 $$ \begin{array}{l}\mathrm{Constant}\kern0.5em \mathrm{in}\mathrm{fection}\kern0.5em \mathrm{pressure}:\kern0.5em  ln\left\{h(t)\right\}\kern0.5em =\kern0.5em {\beta}_0\\ {}\mathrm{Decrease}\kern0.5em \mathrm{in}\kern0.5em \mathrm{in}\mathrm{fection}\kern0.5em \mathrm{pressure}:\kern0.5em  ln\left\{h(t)\right\}\kern0.5em =\kern0.5em {\beta}_0\kern0.5em -\kern0.5em \beta t\\ {}\mathrm{Periodic}\kern0.5em \mathrm{in}\mathrm{fiction}\kern0.5em \mathrm{pressure}:\kern0.5em  ln\left\{h(t)\right\}\kern0.5em =\kern0.5em {\beta}_0\kern0.5em -\kern0.5em \beta sin\left(2\pi t\right)\end{array} $$

where the infection pressure (*h*) at age *t* is given by the amplitude (*β*) by which the baseline number of parasites (*β*_*0*_) varies, or does not, in a year.

### Models assessing age-dependent infection pressure

The models that assume the existence of an age-dependent infection pressure include a parameter (*ϕ*) representing the reduction in the number of parasites that foxes acquired after exposure which is proportional to the increase of host age. Thus, the infection pressure at age *t* where there is both periodic infection pressure and a decrease with age is given by equation 4:

4 $$ \mathrm{Periodic}\kern0.5em \mathrm{with}\kern0.5em \mathrm{age}\hbox{-} \mathrm{decrease}\kern0.5em \mathrm{in}\kern0.5em \mathrm{in}\mathrm{fection}\kern0.5em \mathrm{pressure}:\kern0.5em  ln\left\{h(t)\right\}\kern0.5em =\kern0.5em {\beta}_0\kern0.5em -\kern0.5em \beta sin\left(2\pi t\right)\kern0.5em -\kern0.5em \phi t $$

where *h* is the infection pressure in number of parasites per year, *β*_*0*_ is the baseline number of parasites in a year, *β* is the amplitude by which this baseline varies periodically and *ϕ* is the decrease in the number of parasites related with fox age.

### Model fitting

*Echinococcus multilocularis* follows a highly aggregated distribution within the animal hosts, thus, we used the negative binomial likelihood function to fit age-based abundance models to the observed data (equation 5).

5 $$ \Pr \left(Z(t)\kern0.5em =\kern0.5em s\right)\kern0.5em =\kern0.5em \frac{\varGamma \left(k\kern0.5em +\kern0.5em s\right)}{\varGamma (k)\kern0.5em s!}{\left(\frac{M}{k\kern0.5em +\kern0.5em M}\right)}^s{\left(\frac{k}{k\kern0.5em +\kern0.5em M}\right)}^k $$

where the probability of the number of parasites (*s*) for each sample (*Z*) at age (*t*) is given by the mean number of parasites (*M*) predicted by the model, where Γ represents the gamma distribution and *k* is the negative binomial constant of aggregation. The values for the aggregation constants for each spatial zone were estimated from the observed data using the *glm.nb* function from the MASS package in R [35]. In addition, we assumed a common negative binomial constant for all age groups, as it has been previously reported [31]. We explored variable aggregation between the zones by assigning different values of *k* to each zone.

The life expectancy of the parasite (1/*μ*) was estimated from the model fit, allowing *μ* to be data driven. This was compared to an estimate of *μ* of 8.6 from the data presented in Kapel et al. [36]. Equations (1) and (2) including any variation in *h* over time, as described by equation (3), were numerically integrated using the ode function in the deSolve package in R [37].

Based on this probability model, a likelihood function was computed stating the probability to observe the data, given the model. The transformed, negative log-likelihood (NLL) function was minimized using the *optim* function of the statistical package in R [38]. All R code is provided in Additional file 2.

For model comparison and selection we followed the method described in Rüegg et al. [39]. The NLL of each competing model was plotted against the number of parameters. This method provided a visual aid to identify the best fitting models for each number of parameters. The selected models were then compared in pairs in increasing order of complexity, starting with the simplest model (M1). The difference of NLL between each pair of models was tested against an empirical probability distribution of the null hypothesis that the simpler model provides a better fit to the data. To this end, 500 populations were simulated from the simpler model. For each population the two competing models were fitted and the difference in NLL was computed. The NLL difference estimated from the data was then compared to the 95%-quantile of this distribution. Therefore, the more complex model would give a better fit just by chance in less than 5% of the cases (*α* = 0.05).

### Parameter estimation

Key epidemiological parameters were quantified from the best fitting model and confidence intervals were estimated by bootstrapping. We generated 1,000 data sets and estimated the parameter values by resampling with replacement from the originally data set. That is creating 1,000 data sets, each of 531 data points being the size of the original sample of data. For the confidence interval, we reported the 2.5th and 97.5th percentiles of the bootstrap samples. For the confidence bands of the most parsimonious model, these 1,000 data sets were used to generate predicted abundances at each time point to then report their 2.5^th^ and 97.5^th^ percentiles.

### Number of parasites per infectious insult

Using the results of the model quantifying the force of infection with the same data set [25], we estimated the numbers of parasites per infectious insult acquired by foxes in the periurban and urban zone at times of highest and lowest infection pressure, by using the simple equation:

$$ Number\kern0.5em  of\kern0.5em  parasites\kern0.5em  per\kern0.5em  infectious\kern0.5em  insult\kern0.5em =\kern0.5em \frac{Infection\kern0.5em  pressure\kern0.5em \left( parasites\kern0.5em  per\kern0.5em  unit\kern0.5em  time\right)}{Force\kern0.5em  of\kern0.5em  infection\kern0.5em \left( insults\kern0.5em  per\kern0.5em  unit\kern0.5em  time\right)} $$

Complete analysis of the data was performed using the open source software in R [38].

## Results

### Exploratory analysis

The exploratory analysis of the data showed that foxes aged up to 3 years old, which represented 86% of the total samples, accounted for 88% of all infected animals and harboured 94% of the total parasite biomass. The study data encompassed 531 observations categorised by age of the host (juveniles, *n* = 309; adults between 1 and 2 years, *n* = 99; adults between 2 and 3 years, *n* = 50; and adults over 3 years, *n* = 73), type of urbanization zone (periurban, *n* = 185; border, *n* = 200; and urban, *n* = 146) and season when the fox was collected (spring, *n* = 31; summer, *n* = 123; autumn, *n* = 113; and winter, *n* = 264). The seasons were defined in groups of three months: spring (March to May), summer (June to August), autumn (September to November) and winter (December to February).

The parasite counts varied widely between observations with no parasites in 59% of the foxes, 21% foxes found with 1–99 worms, 17% foxes with 100–9,999 worms, and 3% of them with more than 10,000 worms. The proportion of parasite loads found in the foxes by type of urbanization zone, fox age and season are displayed in Table 2.

**Table 2:** Observed proportions of *E. multilocularis* abundance (number of parasites/total number of parasites retrieved) in foxes in Zurich by type of urbanization zone, seasons and fox age

	Fox age (years)				
	< 1	1 to 2	2 to 3	> 3	Total
By Zone					
Periurban	0.51	0.02	0.01	0.04	0.58
Border	0.15	0.05	0.01	0.01	0.22
Urban	0.12	0.08	2e^-4^	0	0.20
Total	0.78	0.15	0.02	0.05	1.00^a^
By Season					
Spring	4e^-3^	1e^-5^	0	0.01	0.01
Summer	0.08	2e^-5^	1e^-5^	5e^-5^	0.08
Autumn	0.16	1e^-3^	4e^-3^	2e^-3^	0.17
Winter	0.53	0.16	0.01	0.05	0.74
Total	0.78	0.16	0.01	0.06	1.00^a^

^a^Total number of parasites retrieved = 534,815 parasites

The distribution of *E. multilocularis* in foxes was highly aggregated (overall negative binomial constant *k* = 0.05).

### Model comparison

**Fig. 2:**
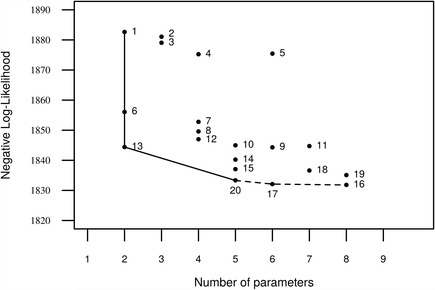
Model comparison for *E. multilocularis* abundance models in foxes in Zurich. Model performance is assessed based on the smallest negative log-likelihood (NLL) for a given number of parameters used. Starting with the simplest model (Model 1), models along the lower left edge of the cloud (Models 1, 6, 13, 20, 17 and 16) were selected and compared pair-wise. Significant differences are shown as full line, while comparisons with results that did not have statistically significant differences are broken lines

Transmission models comparing the possibility of acquired immunity or changes in infection pressure were compared to explore the hypotheses whether parasite induced immunity, seasonality, spatial differences and host age may be contributing to the observed pattern of parasite abundance in the foxes. A total of 20 models describing different scenarios for parasite transmission were compared based on their goodness-of-fit to the data and the number of parameters used, as it is illustrated in Fig. 2. The best fitting model was M20 (Table 3). Thus, models M1-M5 which described no spatial variation in transmission and models M6-M12 in which there were 2 spatial zones of transmission had a poorer fit to the data generally than models M13 to M20 where there were three spatial zones. Of these latter models those with a periodic infection pressure (M15-M20) described the data better than a non-periodic infection pressure (M13-M14). M20 and M19, with a decreasing abundance only in the periurban zone described the data better than M15 and M16 with either no decrease in infection pressure in any zone or a decrease in all 3 zones. M20 where the lower abundance in old foxes in the periurban zone is best explained by decreasing infection pressure in old fox gives a better description of the data than M19 where is hypothesizes it is due to parasite induced immunity. The difference between M17 and M20 is fixing the life expectancy of the parasite to that experimentally observed (M20) rather than using the data.

**Table 3:** Description and goodness-of-fit results of the abundance models for *E. multilocularis* in foxes in Zurich

Model	Description	P	NLL
One zone			
M1	Constant infection pressure	2	1,882.6
M2	Decrease in infection pressure and fox age	3	1,881.1
M3	Periodic relationship between infection pressure and fox age	3	1,879.1
M4	Periodic relationship between infection pressure and fox age plus a decreasing infection pressure with increasing fox age	4	1,875.3
M5	Periodic relationship between infection pressure and fox age plus a decreasing infection pressure with increasing fox age and also accounting for parasite-induced immunity	6	1,875.4
Two zones: periurban and suburban (border + urban)			
M6	Constant infection pressure	2	1,856.1
M7	Decrease in infection pressure and fox age	4	1,852.8
M8	Periodic relationship between infection pressure and fox age	4	1,849.6
M9	Periodic relationship between infection pressure and fox age plus a decreasing infection pressure with increasing fox age in both zones	6	1,844.3
M10	Periodic relationship between infection pressure and fox age plus a decreasing infection pressure with increasing fox age only in periurban zone	5	1,845.0
M11	Periodic relationship between infection pressure in both zones and only in the periurban area decreasing infection pressure with increasing fox age and parasite-induced immunity	7	1,844.7
M12	As M10, but *μ* ^a^ was fixed as 8.6	4	1,847.0
	Three zones: periurban, border and urban		
M13	Constant infection pressure	2	1,844.4
M14	Decrease in infection pressure and fox age	5	1,840.2
M15	Periodic relationship between infection pressure and fox age	5	1,837.1
M16	Periodic relationship between infection pressure and fox age plus a decreasing infection pressure with increasing fox age in all three zones	8	1,831.8
M17	Periodic relationship between infection pressure and fox age plus a decreasing infection pressure with increasing fox age only in periurban zone	6	1,832.1
M18	Periodic relationship between infection pressure and fox age plus parasite-induced immunity in all three zones	7	1,836.6
M19	Periodic relationship between infection pressure and fox age in all zones and only in the periurban area decreasing infection pressure with increasing fox age and parasite-induced immunity	8	1,835.1
M20	As M17, but *μ* ^a^ was fixed as 8.6	5	1,833.4

^a^Parasite death rate (*μ*)

*Abbreviations*: *P* model parameters, *NLL* negative log-likelihood values

### Best-fitting model

The best-fitting model, M20, assumed different parasite burdens in foxes from the periurban, border, and urban zones. The estimations of the negative binomial constants indicated variability in the degree of aggregation of the parasites (*k*) between the three zones (*k*_*peri*_ = 0.1, *k*_*border*_ = 0.02, *k*_*urban*_ = 0.05). The model also considered age-dependent infection pressure, but only resulted in a better fit for such a model in the periurban area (i.e. old foxes had a lower exposure rate). In addition, the model suggested the existence of a sinusoidal infection pressure that varied with time with higher peaks during autumn and winter in foxes in all spatial zones, even though this seasonality was highest and most marked in the periurban zone. However, the baseline number of parasites (*β*_*0*_) was found to be similar among the three zones and thus a single *β*_*0*_ was applied to all zones. Finally, the model did not find evidence of parasite-induced immunity. Table 4 gives the maximum likelihood estimates (MLE) of the five parameters estimated by the model. Thus, the infection pressure, as described by equation (4), can be estimated at any time point (*t*) in any spatial zone. For example, a 10-month-old fox in the periurban zone has *β*_*0*_ = 8.5, *βp* = 2.6 and *ϕ* = 0.5 (*t* is in years, so in this case = 0.833). Thus ln[(h(t))] = 8.5 + 2.6*sin(2*π*0.833) -0.5*0.833 = 10.3. Taking the exponent gives an infection pressure (or exposure) of 30,880 parasites per year at that time point. Likewise a 10-month-old fox in the urban zone has *β*_*0*_ = 8.5 and *βu* = 1.2 (*ϕ* = 0 as in model M20) and hence an infection pressure of 13,911 parasites per year.

**Table 4:** Maximum likelihood estimates (MLE) with a negative log-likelihood value of 1,833.4 of the abundance model parameters for *E. multilocularis* in foxes in Zurich with their 95% bootstrap confidence intervals (CI) for Model 20, with μ fixed at 8.6

Parameter	MLE	95% CI	
*β*_*0*_	8.5	7.5–9.3	Baseline number of parasites of the infection pressure
*β*_*p*_	2.6	1.4–4.1	Amplitude of the infection pressure in the periurban zone
*β*_*b*_	0.1	-0.7–1.5	Amplitude of the infection pressure in the border zone
*β*_*u*_	1.2	-1.3–2.7	Amplitude of the infection pressure in the urban zone
*ϕ*_*p*_	0.5	0.3–1.3	Decrease parasite rate in the periurban zone

*Abbreviations*: *MLE* maximum likelihood estimates, *CI* confidence interval

**Fig. 3:**
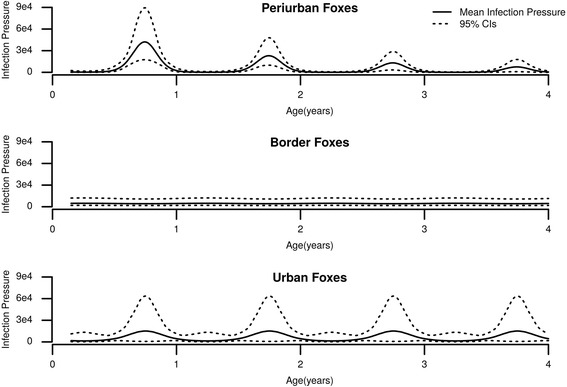
Dynamics of *E. multilocularis* infection pressure (mean and 95% CIs) by fox age in the periurban, border and urban zones predicted by Model 20. The three plots show the variation in infection pressure in foxes by host age up to 4 years old. When fox age is used as a proxy of time the curve peaks correspond to the colder seasons (autumn and winter) separated by flat intervals, which correspond to the warmer seasons (spring and summer)

A graphical representation of the seasonal variation of the infection pressure on each urbanization zone can be found in Fig. 3. The model gives predictions of the infection pressure greater than zero, even for newborn foxes, due to the baseline parameter (*β*_*0*_). However, the fox cubs are not exposed to infection during their lactation period (*c.*4 weeks) thus foxes less than two months were assigned no parasites to their model predictions.

The most parsimonious model (M20) therefore indicated that there were spatial variations in infection pressure, with the periurban area having the highest value of *h*. The infection pressure varied throughout the year in all three spatial zones, with the highest infection pressure occurring in the winter months. Variations in abundance with age that were most notable in the periurban zone were better explained by an age-related decrease in infection pressure rather than prevention of reinfection by immunity resulting from an earlier exposure.

### Parasites per infectious exposure

In periurban foxes, the maximum infection pressure occurred in winter and varied between 36,000 parasites in year 1 (1st winter), 22,000 in year 2 (2nd winter), and 13,000 in year 3 (3rd winter). This results in an approximate mean of 24,000 parasites per fox over the three winters. Lewis et al. [25], using the same data set reported around 9.5 infectious insults per year in winter. Therefore, about 2,500 parasites result from a single infectious insult in periurban foxes during winter. Likewise, in summer periurban foxes are exposed to an average of 230 parasites per year derived from 2.3 insults or 100 parasites per insult.

In urban foxes, we predict an infection pressure during winter of approximately 15,000 parasites per year from 1.8 insults or 8,300 parasites per infection event. In summer infections there are 1,500 parasites per year from 0.5 insults or approximately 3,000 parasites per infection event.

## Discussion

Model 20 was the best fitting model, so the data gives support to the hypotheses that there are: (i) spatial differences in parasite infection pressure among the three zones; (ii) temporal differences in parasite infection pressure in relation to time of the year and; (iii) there are infection pressure variations across different age groups only in the periurban area. These findings are consistent with some of the often interrelated and frequently reported risk factors in EM infection in foxes [40]. Nevertheless, some of the model implications are not in line with previous research. These findings are discussed further below in detail.

First, the model describes spatial differences in infection pressure across urbanization zones. Urban resident foxes in Zurich have been found to display small home ranges (*c.*25 ha) and they pursuit their daily activities mainly within this area, although some movement of foxes among urbanization zones also occurs [41, 42]. The level of urbanization of their limited territories determines the number of rodents and foxes and their predator-prey interactions, influencing ultimately parasite transmission [40]. Therefore, the model hypothesis of an existing heterogeneous distribution of infected foxes within the city is consistent with numerous studies that found an association between infection status and type of urbanization zone [40]. Even though this association has been often linked to other risk factors such as season [11, 16] and fox age [17]. Most of these studies also reported a gradual decrease in parasite prevalence from the rural areas and the periphery of the cities towards the more urbanised zones [27–29]. Similarly, Lewis et al. [25] estimated a higher number of infection exposures in periurban foxes (maximum of 9.35 to 9.7 insults/year) compared to urban foxes (maximum of 1.6 to 2 insults/year) in Zurich. Foxes from the outside and transition areas of the cities seemed to prey more on rodents and hence, be more exposed to parasite infection [17, 29]. This comes as a result of the presence of high densities of suitable intermediate hosts bearing high parasite prevalences in the outskirt of the cities [16, 17]. In contrast, urban foxes rely more on anthropogenic food for their diet, being highly abundant and accessible in the city center [14]. Similarly, our model estimated the highest amplitude to be in the periurban zone, implying that the highest infection pressure was borne by the periurban foxes. However, in our study this is just applicable to juvenile foxes since M20 describes the infection pressure in the periurban area is age-dependent resulting in adult foxes being exposed to less number of parasites per infection insult than their young. Consequently, only the periurban juveniles presented the highest infection pressure across all areas. In fact, the model predictions referring to adult foxes suggested the urban foxes harboured the highest infection pressure among zones. This is a surprising finding since it would be expected that animals living in the edge of the cities would prey more on rodents and thus be more exposed to infection as previously discussed. The model suggests that periurban adults are being infected more frequently on average, but that each infection event results in fewer parasites than a typical infection event in urban foxes. In absence of host immune responses to infection it may indicate that infected rodents that are consumed by urban foxes have greater numbers of protoscolices than those consumed by periurban foxes, even though urban foxes are consuming fewer infected rodents. This hypothesis differs to what has been reported in terms of parasite infection in city foxes [40]. A potential explanation might be that some super infected foxes collected in the urban area were in fact dispersal foxes whose home range encompassed mainly the border area but they died in the urban area during an excursion looking for feeding or mating opportunities. The occurrence of so-called floating individuals with larger home ranges has been previously recorded in Zurich [41]. These foxes are commonly young males that expand their range during the mating season (autumn and winter) [43]. In this case, seasonal variations in the spatial behaviour of foxes could explain the higher amplitude in the infection pressure found in the urban area. Alternatively, it could be due to the short history of urban colonization of foxes, suggesting that the transmission cycle is not yet equilibrated, showing typical flickering in transiting complex systems [44]. In any case, there is an increasing individual risk of developing AE mainly in areas where high densities of humans and urban foxes intersect [15, 24], which it is not the case of the city centre. The existence of a high infection pressure in the periphery of the cities and in the transition areas and adjacent spatial zones are still the main cause of concern in terms of AE transmission risk.

Secondly, the model also accounts for a sinusoidal infection pressure throughout the year with peaks during the cold months of autumn and winter. In Zurich, higher infection rates have been previously recorded during winter, in association with the host age and the city zone where the fox was retrieved [11, 16]. Likewise, Lewis et al. [25] found a periodic force of infection with an annual minimum of 0.27–1.27 parasite insults and a maximum of 6.87–7.05 parasite insults per year in foxes collected in Zurich. Evidence of seasonal variation in prevalence in foxes has been frequently reported in other locations [26, 45, 46]. In fact, seasonal fluctuations in temperature and precipitation are often proposed as infection determinants because of their influence on hosts’ numbers and parasite survival in the environment [40]. The low temperatures and humidity favour the survival of *E. multilocularis* eggs in the environment, potentially contributing to the occurrence of higher infection rates during the cold and rainy seasons [47]. In addition, the influence of climatic changes on the abundance and age-structure of vole populations [48–50] may have an impact on fox predation on voles [51] and consequently in the degree of parasite infection in foxes. Some studies have found that foxes showed higher predation on voles in autumn compared to spring coinciding with prey availability [11, 29]. Whereas, it has been reported a correlation between low day temperatures and higher infection rates in rodents [49, 50].

Thirdly, the model suggests the existence of an age-dependent infection pressure was found only in the periurban area. Periurban foxes prey more on rodents compare to other urbanised zones [17], thus if there is any effect of fox age in parasite exposure it is more likely to be evident in this area. In Zurich, higher worm burdens have been recorded in younger foxes compared to adults [11]. In addition, seasonal variations in prevalence were more marked in the juvenile animals in the same Swiss city [17]. Despite being a young fox has been repeatedly reported as an infection determinant for *E. multilocularis* [40], the underlying cause remains unclear. Potential reasons for decreasing parasite abundance in the periurban adult fox may include predatory behaviour or diet preferences. Juveniles might have a higher proportion of rodents in their diet, as they are abundant and easy to prey whereas adult foxes might have better access to more difficult prey or have more experience finding food from anthropogenic sources. Alternatively, inexperienced juveniles might be inclined to prey on infected voles if parasite infection adversely affects the intermediate host [52]. Nevertheless, although variation in feeding behaviour across age groups of foxes has not been demonstrated, it has been hypothesized that juvenile foxes may have best access to voles [17]. The dietary response of red foxes is complex when abundant alternative resources are available such as anthropogenic food and multiple intermediate host species [53]. The diet of urban foxes has a dominance of scavenged meat and other scavenged and cultivated fruit and crops with more than half of an average stomach content being anthropogenic. The proportion of scavenged food recovered from foxes' stomachs increases in foxes found in the city center compared to the periurban area [14, 17]. Such a variation in fox dietary preference correlates with the spatial variations in infection pressure reported in the best fitting model M20.

Other proposed explanation for age-related differences in burdens of parasites considers the existence of a developing immunological response after repeated infection [11, 26, 54]. There are previous studies in highly endemic regions for *E. granulosus* which document a negative correlation of parasite abundance with age in dogs which would be predicted if parasite-induced host immunity limited infection [31, 55, 56]. In addition, experimental infections have shown evidence of parasite-specific antibody responses in animal hosts although it remains unclear their effect on parasite infection [57]. Nonetheless, previous models assuming presence of acquired host immunity did not give a better fit to *E. multilocularis* data in dogs or foxes [25, 55]. Furthermore, studies in Kyrgyzstan and Lithuania failed to demonstrate a decrease in *E. multilocularis* abundance with increasing fox age [58, 59]. Moreover, in our study the parameter values on which the models incorporating immunity converged indicated a very high value of γ – the rate of loss of immunity. This would indicate that the duration of immunity following exposure would only be a matter of weeks at most, and require conditions of extremely high infection pressure to be maintained. Even if immunity were present its effect on parasite abundance would be negligible with this SIR model. Thus, the better fit to data given by the models without immunity or the potential very high rates of loss of immunity, if present, are evidence that definitive host immunity is not regulating the parasite population in this system.

Experimental studies where foxes were artificially infected have reported a pre-patent period of 29–33 days [60] and a patent period of up to three months [61]. In the study of Kapel et al. [36] it took approximately 42 days for foxes experimentally infected with 20,000 protoscolices to reduce their worm load to 50%. We used this measure to calculate the parasite death rate in models M12 and M20 as the data itself was not able to define this parameter well (Table 3).

We have attempted to quantify the infection pressure of *E. multilocularis* in foxes in Zurich to gain a better insight on parasite epidemiology through hypothesis testing using a relatively simple transmission model. The modelling of the *E. multilocularis* infection is potentially a complex task since the dynamics of parasite transmission are influenced by a wide range of interrelated factors such as, hosts’ population densities, predator-prey interactions, landscape characteristics, climate conditions and human-related activities [40]. Additionally, the modelling of parasite abundance brings extra challenges due to the extreme aggregation of parasites within their hosts. This intense aggregation produces a high degree of uncertainty to the model predictions. This is reflected in the wide confidence intervals related to the model predictions. Such wide confidence intervals could have been narrowed if the data set had had more data points. However, despite this the major findings of the study are robust as the most parsimonious model had an improved likelihood (or statistical fit) compared to the competing models (representing competing hypotheses). Furthermore, the basic model was first proposed by Roberts et al. [30] and has since been used on several data sets [31, 56, 62] and has proved robust, despite its relative simplicity. The present study introduced potential seasonal variations in infection pressure by allowing the parameter *h* to have a sinusoidal relationship with age (and hence time). Other data sets analyzed with this model were taken at 1 time point and thus could not analysed in such a way. Thus, in terms of the hypotheses we tested the model appears to have validity and robustness, although it would further support our findings if or when another similar data set becomes available to confirm this.

Nevertheless, models are conceived to be a simplified representation of the highly complex processes in nature and provide a useful tool to assess different hypotheses. All models are wrong, but the question remains as to how wrong the model must be to lose its usefulness [63]. Given the assumptions in the model, the data suggests spatial and age-related variations in infection pressure to foxes. Therefore, even considering all limitations, the model offers a practical platform to improve knowledge on parasite epidemiology and to allow the quantification of epidemiological parameters that cannot be measure directly in the field, such as the infection pressure. Some of the implications derived from the model concurred with previous epidemiological knowledge on *E. multilocularis* infection, such as the existence of spatial heterogeneities, seasonal fluctuations and age-related differences. Alternatively, other conclusions diverged from previous reports, such as finding that the highest number of parasites developing in the fox after infection exposure occurs in the urban area. However, it cannot be ruled out the possibility that the few urban foxes found harbouring high loads of parasites might have become infected in the neighboring area previously to their incursion into the urban zone. The model also challenged the hypothesis that parasite-induced host immunity may play a role in the transmission dynamics of *E. multilocularis*. Using the models described we found no convincing evidence that this may be the case. The decrease in abundance in foxes, only observed in the periurban zone, is better explained by a decrease in infection pressure in older foxes, although the differences in model predictions are quite subtle.

## Conclusions

In conclusion, the model gives a picture of the overdispersed infection pressure borne by foxes in Zurich, highlighting the potentially large contribution of young periurban foxes and foxes from the outside perimeter of urban areas towards environmental contamination. Previous studies have proved the efficacy of the use of anthelmintic baiting to reduce the environmental contamination with parasitic eggs [64–66]. Similarly, in Zurich the placement of monthly baits along the urban periphery has been shown to successfully decrease the amount of coproantigen-positive fox faeces and reduce infection rates in intermediate hosts (*A. terrestris*) in baits areas [67]. However, temporal anthelmintic interventions mostly failed to achieve permanent parasite elimination [64, 68]. Hence, there is a need to ensure the optimisation of potential long-term baiting campaigns [19]. Model results suggest that a reduction in parasite biomass in Zurich foxes could be more effectively achieved if baiting strategies were to be intensified in the periphery of the city and during the autumn and winter months. The quantification of the temporal-spatial variation of the number of parasites in foxes can help to optimise the designing of targeted bait programmes aiming to reduce the level of environmental contamination and ultimately, infection risk in humans.

## Additional files

Additional file 1: Data S1. File containing original data. (XLSX 20 kb)
Additional file 2: Text S2. R code. (DOC 44 kb)

## Abbreviations

AE: Alveolar echinococcosis
CI: confidence intervals
MLE: maximum likelihood estimate
NLL: negative log-likelihood
