# Supplementary material for: Mathematical modelling of Echinococcus multilocularis abundance in foxes in Zurich, Switzerland
Source: Parasit Vectors. 2017 Jan 11;10:21. doi: 10.1186/s13071-016-1951-1 (PMC5225524; doi:10.1186/s13071-016-1951-1)
Supplement: Additional file 2: Text S2. — R code. (DOC 44 kb) [file 13071_2016_1951_MOESM2_ESM.doc]

**Additional file 2.** R Code

**1. Estimating parameters**

library(deSolve)

library(MASS)

#load the data

abunP<-read.csv(“abundP.csv”, header=T)) #periurban data

abunB<-read.csv(“abunB.csv”, header=T)) #border data

abunU<-read.csv(“abunU.csv”, header=T) #urban data

# periurban zone- differential equation for model 20

ech1<-function(t, state, parms) {

with(as.list(c(state, parms)), {

dM <- exp(-x*t)*exp(b0-b*sin(2*pi*t))-8.625*M

return(list(c( dM)))

})

}

state <- c(M=0)

# border zone - differential equation for model 20

ech2<-function(t,state,parms){

with(as.list(c(state,parms)),{

dMbor<-exp(b0-bbor*sin(2*pi*t))-8.625*Mbor

return(list(c(dMbor)))

})

}

statebor <- c(Mbor=0)

# urban zone- differential equation for model 20

ech3<-function(t, state, parms) {

with(as.list(c(state, parms)), {

dMurb <- exp(b0-burb*sin(2*pi*t))-8.625*Murb

return(list(c( dMurb)))

})

}

stateurb <- c(Murb=0)

#inserts a first row of 0 counts at time=0 to allow initiating numerical integration.

initb=data.frame(Age_days=0, Zone="border", Abun=0, years=0)

abunB<-rbind(initb, abunB)

initp=data.frame(Age_days=0, Zone="periurban", Abun=0, years=0)

abunP<-rbind(initp, abunP)

initu=data.frame(Age_days=0, Zone="urban", Abun=0, years=0)

abunU<-rbind(initu, abunU)

#likelihood function

pred20 <- function(parms) {

out1 <- ode(y = state, func = ech1, parms = parms, times = abunP$years)

neglogkp<- -sum(dnbinom(x=abunP$Abun,size= 0.09758278, mu=out1[,"M"], log = TRUE)) # periurban

out2 <- ode(y = statebor, func = ech2, parms = parms, times = abunB$years)

neglogkb<- -sum(dnbinom(x=abunB$Abun, size=0.05246528, mu=out2[,"Mbor"], log = TRUE)) # border

out3 <- ode(y = stateurb, func = ech3, parms = parms, times = abunU$years)

neglogku<- -sum(dnbinom(x=abunU$Abun, size=0.01560734, mu=out3[,"Murb"], log = TRUE)) # urban

if(is.infinite(neglogkp)){return(1e7)}

if(is.infinite(neglogkb)){return(1e7)}

if(is.infinite(neglogku)){return(1e7)}

tot=neglogkp+neglogkb+neglogku

return(tot)

}

#loading starting parameters

start<-c(x=0.5, b0=8,b=2,burb=2, bbor=2)

#Minimizing the negative log likelihood

fit1<- optim(par=start,

fn=pred20, control=c(maxit=10000))

# periurban immunity – alternative differential equation for model 19 to substitute in likelihood function.

ech1<-function(t, state, parms) {

with(as.list(c(state, parms)), {

dS <- g*(1-S)-a*(exp(-x*t)*exp(b0-b*sin(2*pi*t)))*S

dM <- (exp(-x*t)*exp(b0-b*sin(2*pi*t)))*S - muu*M

return(list(c(dS,dM)))

})

}

state <- c(S=1, M=0)

start<-c(x=0.5,b0=8, b=2.6, burb=2, bbor=2,a=1, g=8.2,muu=4)

**2. Bootstrap Cis (model 17)**

library(deSolve)

library(MASS)

# periurban zone- differential equation for model 17

ech1<-function(t, state, parms) {

with(as.list(c(state, parms)), {

dM <- exp(-x*t)*exp(b0-b*sin(2*pi*t))-8.625*M

return(list(c( dM)))

})

}

state <- c(M=0)

# border zone - differential equation for model 17

ech2<-function(t,state,parms){

with(as.list(c(state,parms)),{

dMbor<-exp(b0-bbor*sin(2*pi*t))-8.625*Mbor

return(list(c(dMbor)))

})

}

statebor <- c(Mbor=0)

# urban zone- differential equation for model 17

ech3<-function(t, state, parms) {

with(as.list(c(state, parms)), {

dMurb <- exp(b0-burb*sin(2*pi*t))-8.625*Murb

return(list(c( dMurb)))

})

}

stateurb <- c(Murb=0)

#state initial bounds

start=c(b=2.56,b0=8.45 , x=0.53,burb=1.20,bbor=0.13)

#Minimizing the negative log likelihood

fit1<- optim(par=start,

fn=pred20, control=c(maxit=10000))

#Save results

newres<-as.data.frame(fit1$par)

mle1<-as.data.frame(fit1$value)

for(i in 1:1000) {

#bootstrap new data set for periurban zone

newP<-abunP[sample(1:nrow(abunP), 185, replace=TRUE),]

newP1<-newP[order(newP$years),]

initp=data.frame(Age_days=0, Zone="periurban", Abun=0, years=0)

newP1<-rbind(initp, newP1)

pk<-glm.nb(newP$Abun~1)$theta

#bootstrap new data set for Border zone

newB<-abunB[sample(1:nrow(abunB), 200, replace=TRUE),]

newB1<-newB[order(newB$years),]

initb=data.frame(Age_days=0, Zone="border", Abun=0, years=0)

newB1<-rbind(initb, newB1)

bk<-glm.nb(newB$Abun~1)$theta

#bootstrap new data set for urban zone

newU<-abun U[sample(1:nrow(abunU), 146, replace=TRUE),]

newU1<-newU[order(newU$years),]

initu=data.frame(Age_days=0, Zone="urban", Abun=0, years=0)

newU1<-rbind(initu, newU1)

uk<-glm.nb(newU$Abun~1)$theta

}

#three populations - calculating the likelihood - predicted abundance given parameters

pred10 <- function(parms) {

out1 <- ode(y = state, func = ech1, parms = parms, times = newP1$years)

neglogkp<- -sum(dnbinom(x=newP1$Abun,size=pk, mu=out1[,"M"], log = TRUE)) #periurban

out2 <- ode(y = statebor, func = ech2, parms = parms, times = newB1$years)

neglogkb<- -sum(dnbinom(x=newB1$Abun,size=bk, mu=out2[,"Mbor"], log = TRUE)) #border

out3 <- ode(y = stateurb, func = ech3, parms = parms, times = newU1$years)

neglogku<- -sum(dnbinom(x=newU1$Abun,size=uk, mu=out3[,"Murb"], log = TRUE)) # urban

if(is.infinite(neglogkp)){return(1e7)}

if(is.infinite(neglogkb)){return(1e7)}

if(is.infinite(neglogku)){return(1e7)}

tot=neglogkp+neglogkb+neglogku

return(tot)

}

#Minimizing the negative log likelihood

fit10<- optim(par=fit10$par,

fn=pred10, control=c(maxit=10000))

#Save results

newres<-cbind(newres, as.data.frame(fit1$par)) #appends the results of each iteration

mle1<-cbind(mle1, as.data.frame(fit1$value)) #appends the results of NegLogLkh each iteration

**3. Estimating Confidence bands and plotting**

library(matrixStats)

times1<-seq(0.15, 4, by=0.01) #for plots from 2 months of age

#periurban

hres<- function (t){

h<-exp(-x*t)*exp(b0-b*sin(2*pi*t))

return (h)

}

b0=newres[2,1]

b=newres[3,1]

burb=newres[4,1]

bbor=newres[5,1]

x=newres[1,1]

rest<-as.data.frame(hres(times1))

rest1<-rest

for (i in 2:1000){

b0=newres[2,i]

b=newres[3,i]

x=newres[1,i]

rest<-as.data.frame(hres(times1))

rest1<-cbind(rest1, rest)

}

bandsP<-rowQuantiles(as.matrix(rest1), probs=c(0.025,0.5,0.975))

#border

hres2<-function (t){

hb<-exp(b0-bbor*sin(2*pi*t))

return(hb)

}

b0=newres[2,1]

bbor=newres[5,1]

rest<-as.data.frame(hres2(times1))

restB<-rest

for (i in 2:1000){

b0=newres[2,i]

bbor=newres[5,i]

rest<-as.data.frame(hres2(times1))

restB<-cbind(restB, rest)

}

bandsB<-rowQuantiles(as.matrix(restB), probs=c(0.025,0.5,0.975))

#urban

hres3<-function (t){

hu<-exp(b0-burb*sin(2*pi*t))

return(hu)

}

b0=newres[2,1]

burb=newres[4,1]

rest<-as.data.frame(hres3(times1))

restU<-rest

for (i in 2:1000){

b0=newres[2,i]

burb=newres[4,i]

rest<-as.data.frame(hres3(times1))

restU<-cbind(restU, rest)

}

bandsU<-rowQuantiles(as.matrix(restU), probs=c(0.025,0.5,0.975))

##Infection pressure 3 zones

tiff(file="allzones.tiff", compression="lzw",res=600, width=10, height=3, units="in")

par(mfrow=c(3,1),mar=c(8,8,2,4))

#periurban

lablist.y<-as.vector(c("0", "3e4", "6e4", "9e4"))

lablist.x<-as.vector(c("0", "1", "2", "3", "4"))

plot(times1, bandsP[,2], ylim=c(0, 90000), type="l", lwd=2, axes=FALSE, xlab="", ylab="")

axis(2, at=seq(0,90000, by =30000), labels=FALSE)

axis(1, at=seq(0,4, by=1), labels=FALSE)

text(y=seq(0, 90000, by=30000), par("usr")[1],labels=lablist.y, pos=2, offset=1, xpd=TRUE, cex=0.6)

text(x=seq(0, 4, by=1), par("usr")[1],labels=lablist.x, pos=1, offset=1, xpd=TRUE, cex=0.6)

title(xlab="Age(years)", line=1.5, cex.lab=0.8)

title (ylab="Infection Pressure", line=2, cex.lab=0.8)

lines(times1,bandsh[,1],col="red",lwd=1)

lines(times1,bandsh[,3],col="red",lwd=1)

mtext("Periurban Foxes", cex=0.8, font=1,line=-8, side=1.5)

#border

lablist.y<-as.vector(c("0", "3e4", "6e4", "9e4"))

lablist.x<-as.vector(c("0", "1", "2", "3", "4"))

plot(times1, bandsB[,2], ylim=c(0, 90000), type="l", lwd=2, axes=FALSE, xlab="", ylab="")

axis(2, at=seq(0,90000, by =30000), labels=FALSE)

axis(1, at=seq(0,4, by=1), labels=FALSE)

text(y=seq(0, 90000, by=30000), par("usr")[1],labels=lablist.y, pos=2, offset=1, xpd=TRUE, cex=0.6)

text(x=seq(0, 4, by=1), par("usr")[1],labels=lablist.x, pos=1, offset=1, xpd=TRUE, cex=0.6)

title(xlab="Age(years)", line=1.5, cex.lab=0.8)

title (ylab="Infection Pressure", line=2, cex.lab=0.8)

lines(times1,bandsB[,1],col="red",lwd=1)

lines(times1,bandsB[,3],col="red",lwd=1)

mtext("Border Foxes", cex=0.8, font=1,line=-8, side=1.5)

#urban

lablist.y<-as.vector(c("0", "3e4", "6e4", "9e4"))

lablist.x<-as.vector(c("0", "1", "2", "3", "4"))

plot(times1, bandsU[,2], ylim=c(0, 90000), type="l", lwd=2, axes=FALSE, xlab="", ylab="")

axis(2, at=seq(0,90000, by =30000), labels=FALSE)

axis(1, at=seq(0,4, by=1), labels=FALSE)

text(y=seq(0, 90000, by=30000), par("usr")[1],labels=lablist.y, pos=2, offset=1, xpd=TRUE, cex=0.6)

text(x=seq(0, 4, by=1), par("usr")[1],labels=lablist.x, pos=1, offset=1, xpd=TRUE, cex=0.6)

title(xlab="Age(years)", line=1.5, cex.lab=0.8)

title (ylab="Infection Pressure", line=2, cex.lab=0.8)

lines(times1,bandsU[,1],col="red",lwd=1)

lines(times1,bandsU[,3],col="red",lwd=1)

mtext("Urban Foxes", cex=0.8, font=1,line=-8, side=1.5)

dev.off()
